# Ocular size and shape in lens-induced Myopization in young Guinea pigs

**DOI:** 10.1186/s12886-019-1109-y

**Published:** 2019-05-03

**Authors:** Li Dong, Xu Han Shi, Yi Kun Kang, Wen Bin Wei, Ya Xing Wang, Jost B. Jonas

**Affiliations:** 1 Beijing Tongren Eye Center, Beijing Key Laboratory of Intraocular Tumor Diagnosis and Treatment, Beijing Ophthalmology & Visual Sciences Key Lab, Beijing Tongren Hospital, Capital Medical University, Beijing, China; 2 Department of Oncology, Beijing Chao-Yang Hospital, Capital Medical University, Beijing, China; 3 Beijing Institute of Ophthalmology, Beijing Key Laboratory of Ophthalmology and Visual Sciences, Beijing Tongren Eye Center, Beijing Tongren Hospital, Capital Medical University, Beijing Ophthalmology & Visual Science Key Lab, Beijing, China; 4 Department of Ophthalmology, Medical Faculty Mannheim of the Ruprecht-Karls-University Heidelberg, Mannheim, Germany

**Keywords:** Axial length, Globe diameter, Eye diameter, Eye size, Myopia, High myopia, Ocular elongation, Refractive error, Lens-induced axial elongation

## Abstract

**Background:**

Lens-induced myopization in guinea pigs has been used as model for the process of myopization in humans. It has not been explored yet whether the change in globe shape in eyes undergoing myopization is similar in experimental myopia in guinea pigs and in clinical myopia in patients.

**Methods:**

The study included 70 guinea pigs (age:2–3 weeks) equally divided into a study group with lens-induced myopization for 5 weeks, and a control group wearing goggles with no refractive power. The globe diameters were measured using a microcaliper after enucleation.

**Results:**

The horizontal globe diameter (9.19 ± 0.15 mm versus 9.15 ± 0.18 mm; *P* = 0.25) and vertical globe diameter (9.02 ± 0.11 mm versus 8.99 ± 0.14 mm; *P* = 0.29) did not differ significantly between the study group and control group. The sagittal diameter was significantly longer in the study group (8.96 ± 0.15 mm versus 8.84 ± 0.14 mm; *P* = 0.001). While the vertical and horizontal globe diameters were correlated with each other in a ratio of approximately 1:1 (non-standardized regression coefficient B:0.94;95% confidence interval (CI):0.73,1.15), the steepness of the regression lines of the associations of both diameters with the sagittal diameter were flatter (horizontal to sagittal diameter: B: 0.64; 95% CI: 0.44,0.83; vertical to sagittal diameter:B:0.55;95% CI:0.41,0.69). Correspondingly, the ratios of horizontal-to-sagittal globe diameter and of vertical-to-sagittal globe diameter decreased (*P* < 0.001) with longer sagittal diameter.

**Conclusions:**

For each mm axial elongation in young guinea pigs the horizontal globe diameter increased by 0.64 mm (95%CI:0.44,0.83) and the vertical diameter by 0.55 mm (95% CI:0.41,0.69), indicating that the globe enlargement occurred predominantly in the sagittal direction. Axial elongation in guinea pigs led to a similar relative change in ocular shape as in humans.

## Background

Studies on human enucleated eyes and intravital measurements of the globe diameters by magnetic resonance imaging have revealed that with increasing axial length the shape of the globes changed from a spherical form to a prolate configuration [1–3]. In eyes with an axial length of equal to or less than 24 mm, the horizontal globe diameter and the vertical globe diameter increased by 0.44 mm and 0.51 mm, respectively, for each millimeter increase in axial diameter, while in eyes with an axial length of more than 24 mm, the horizontal diameter and the vertical diameter increased by 0.19 mm and 0.21 mm for each millimeter enlargement in axial diameter [3]. These measures agreed with the concept of a mostly axial enlargement of myopic eyes, and that a higher degree of myopic elongation of the eyes was associated with a decreasing enlargement of the eyes in the coronal plane [1–3]. It suggested that the globe enlargement took place predominantly by an elongation of the globe walls close to the equator [4].

For long, the development of myopia in humans has been examined in experimental studies on animals [5–7]. These investigations used several techniques, including the model of lens-induced myopization in guinea pigs [8–15]. Most of these studies however did not explore whether the commonly applied lens-induced myopization in guinea pigs led to a similar change in the globe shape as it has been observed in human eyes with axial myopia. Knowledge about the change in the ocular shape in experimental myopia is important for the discussion whether and how far results from experimental myopia studies can be transferred to human myopic eyes. We therefore conducted this study to assess the relationship of axial elongation to elongation of the coronal diameters in guinea pigs undergoing lens-induced myopization.

## Methods

The experimental study included a total of 70 healthy male pigmented guinea pigs (*Cavia porcellus*) with an age of 2–3 weeks and a body weight of 100–150 g at baseline. The Ethics Committee of the Beijing Tongren Hospital approved the study, and the ARVO Statement and the ARRIVE Guidelines for the use of animals in ophthalmic and vision research were taken into account. The animals were obtained from the Fang Yuanyuan farm, Beijing, China. The total study population was randomly divided into a study group of 35 animals and a control group of 35 animals. The estimation of the sample size of 70 animals for this study was based on the experiences made in previous investigations with a similar study design and also applying the model of lens-induced axial elongation, in which a similar total sample size of about 70 animals was associated with significant differences in axial elongation between a study group and control group.

At start of the study, we glued goggles (refractive power: − 10 diopters, diameter: 15 mm, optical zone: 12 mm) onto the orbital rim of right eyes of the guinea pigs in the study group. The animals of the control group wore the goggles with no refractive power in front of their right eyes for 5 weeks. The goggles were wrapped with a layer of adhesive tape and were attached to the skin which had been cleaned and shaved. Care was taken that the guinea pigs could open their eyes and blink freely under the goggles. The goggles were examined daily to ensure they were clean and in place, otherwise they were detached, cleaned, and re-attached. All guinea pigs were kept at constant temperature of 26 °C. The circadian day/night rhythm was 12 h/12 h (automatically changed at 8 am and 8 pm) with a luminous intensity of 500 Lux.

Five weeks after baseline, all animals were sacrificed by an intraperitoneal injection of an overdose of pentobarbital sodium. The right eyes were enucleated after marking the 12 o’clock position of the eyes. The orbital fat and fascia tissues adherent to the globe were gently removed. At once after that, the sagittal, vertical, and horizontal diameters of the globes were measured using a microcaliper without pressing the globes. The measurements were rounded to the next 0.01 mm.

For statistical analysis, we used a commercially available statistical analysis program (SPSS, version 25.0, IBM-SPSS, Chicago, IL, USA). We first calculated the mean and standard deviation of the diameter measurements and we calculated the ratios of the diameters to each other. We then compared the diameters and the ratios with each other within both groups and between the groups. We finally carried out a linear regression analysis of the associations between the globe diameters.

## Results

The study included 70 globes from 70 guinea pigs. The horizontal globe diameter did not differ significantly between the study group and the control group (9.19 ± 0.15 mm versus 9.15 ± 0.18 mm; *P* = 0.25) nor did the vertical globe diameters (9.02 ± 0.11 mm versus 8.99 ± 0.14 mm; *P* = 0.29). The sagittal diameter was significantly longer in the study group (8.96 ± 0.15 mm versus 8.84 ± 0.14 mm; *P* = 0.001).

**Fig. 1:**
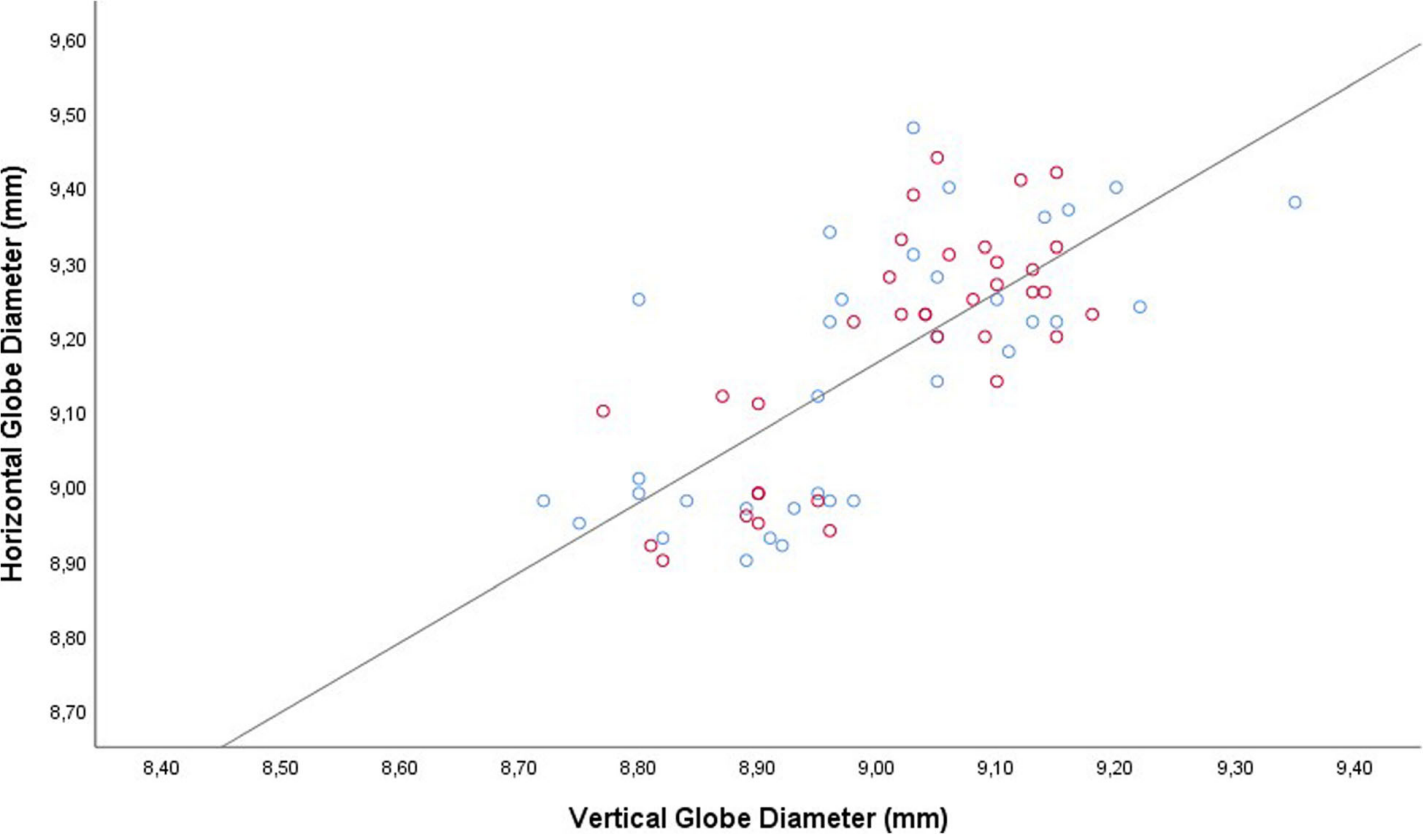
Scattergram showing the correlation between the vertical globe diameter and horizontal globe diameter in young guinea pigs undergoing unilateral lens-induced myopization in the right eyes (study group), with the left eyes remaining untouched (control group); equation of the regression line: Horizontal Diameter (mm) = 0.94 x Vertical Diameter (mm) + 0.73; regression coefficient r^2^ = 0.54; *P* < 0.001. Blue circles: Control group; Red circles: Myopia group

**Fig. 2:**
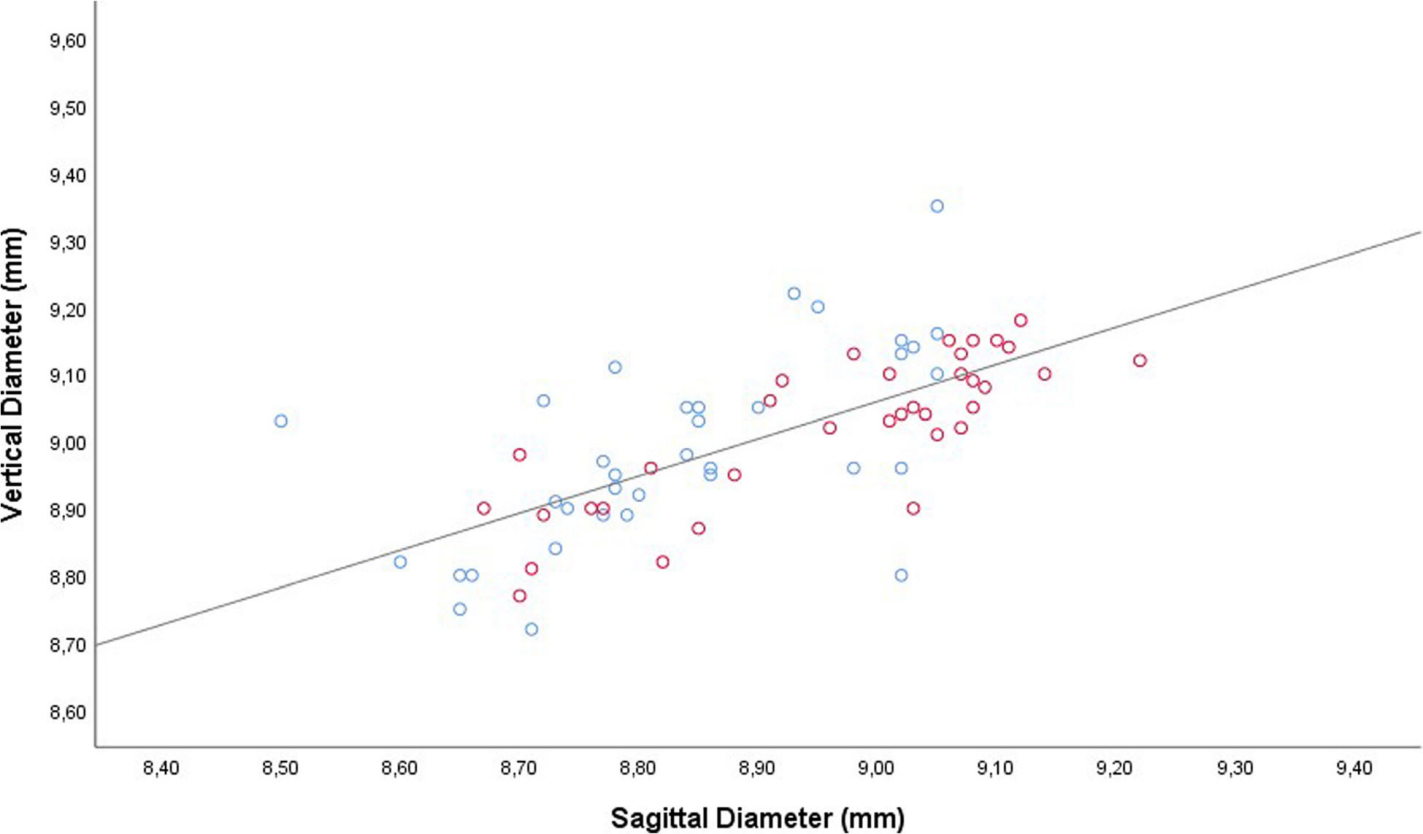
Scattergram showing the correlation between the sagittal globe diameter and vertical globe diameter in young guinea pigs undergoing unilateral lens-induced myopization in the right eyes (study group), with the left eyes remaining untouched (control group); equation of the regression line: Vertical Diameter (mm) = 0.55 x Sagittal Diameter (mm) + 4.08; regression coefficient *r*^2^ = 0.48; *P* < 0.001. Blue circles: Control group; Red circles: Myopia group

**Fig. 3:**
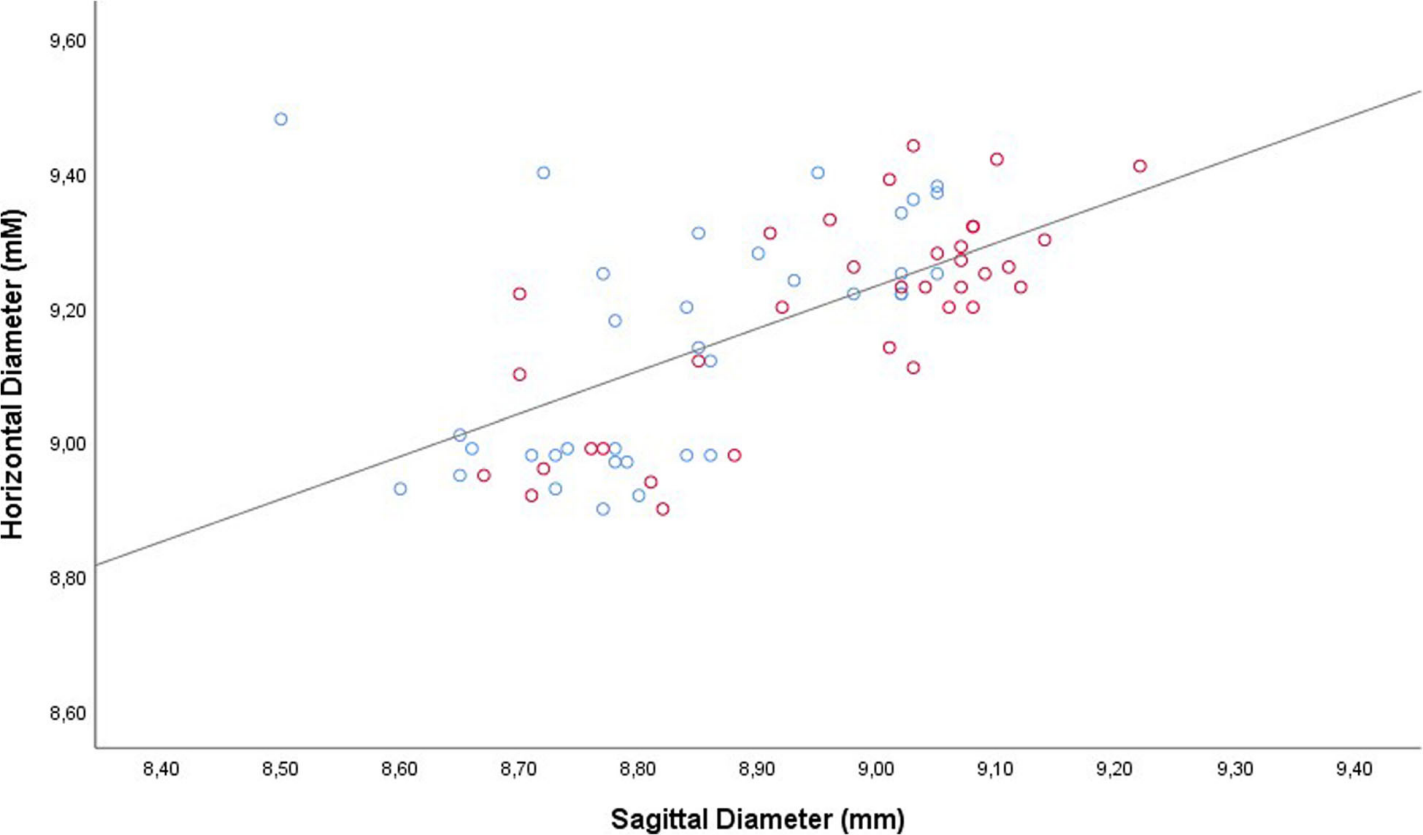
Scattergram showing the correlation between the sagittal globe diameter and horizontal globe diameter in young guinea pigs undergoing unilateral lens-induced myopization in the right eyes (study group), with the left eyes remaining untouched (control group); equation of the regression line: Vertical Diameter (mm) = 0.64 x Sagittal Diameter (mm) + 3.52; regression coefficient r^2^ = 0.38; *P* < 0.001. Blue circles: Control group; Red circles: Myopia group

The vertical globe diameter was significantly correlated with the horizontal globe diameter in the total study population (Fig. 1), and examined separately in the study group and control group, without a major difference in the steepness of the regression line between the study group (non-standardized regression coefficient B: 1.05; 95% confidence interval (CI): 0.73, 1.37) and the control group (B: 0.86 (95% CI: 0.56, 1.16). In the total study population, the steepness of the regression line was 0.94 (95% CI: 0.73, 1.15). Both, the vertical globe diameter and the horizontal globe diameter, were significantly associated with the sagittal diameter (Figs. 2, 3). For both associations, the non-standardized regression coefficients were lower than for the association between the horizontal globe diameter and vertical globe diameter (horizontal to sagittal diameter, B: 0.64 (95% CI: 0.44, 0.83); vertical to sagittal diameter, B: 0.55 (95% CI: 0.41, 0.69; versus horizontal to vertical diameter: B: 0.94 (95% CI: 0.73, 1.15)).

**Fig. 4:**
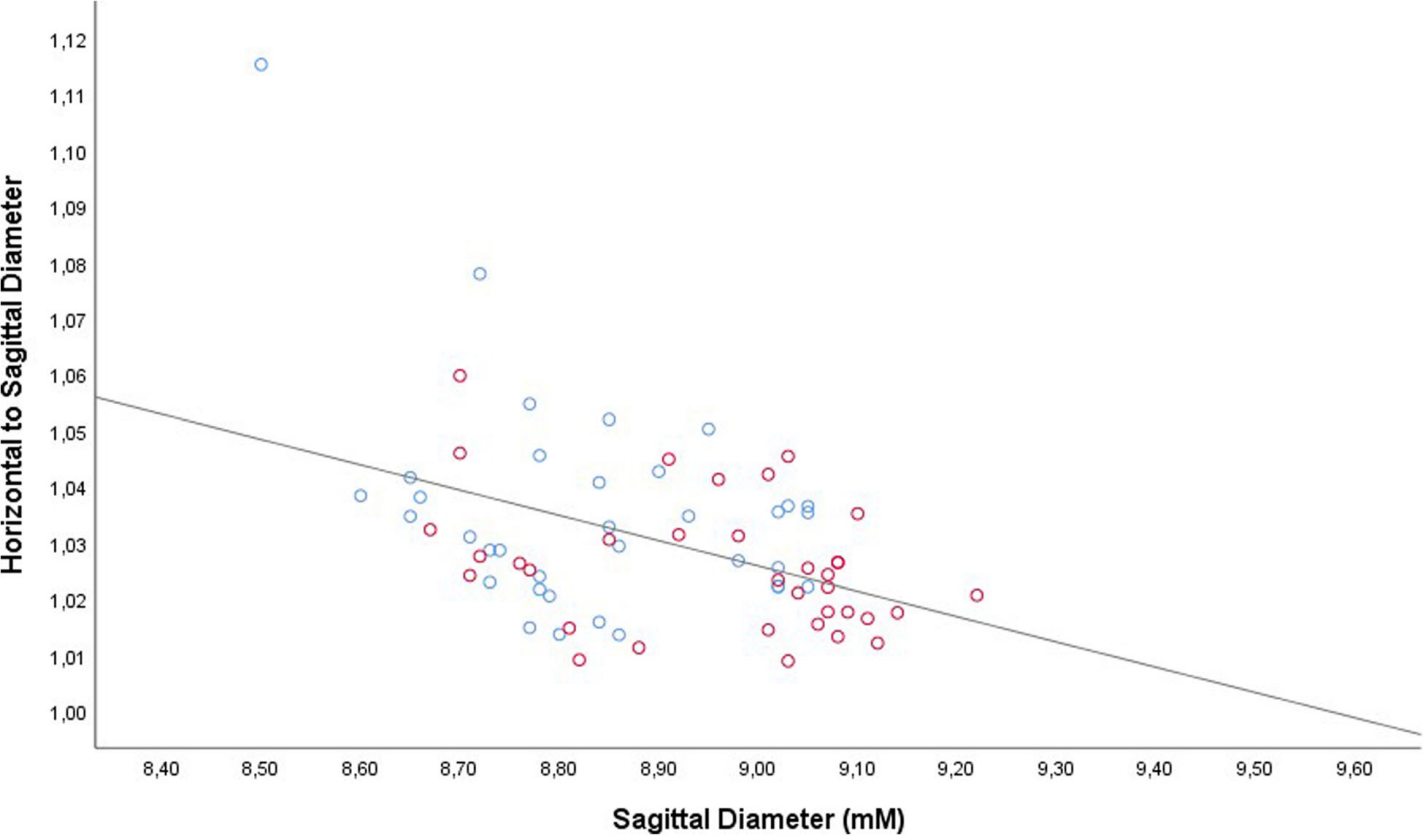
Scattergram showing the correlation between the sagittal globe diameter and the ratio of the horizontal to sagittal globe diameter in young guinea pigs undergoing unilateral lens-induced myopization in the right eyes (study group), with the left eyes remaining untouched (control group); equation of the regression line: Ratio of Horizontal to Sagittal Globe Diameter = − 0.05 x Sagittal Diameter (mm) + 1.43; regression coefficient r^2^ = 0.18; *P* < 0.001. Blue circles: Control group; Red circles: Myopia group

**Fig. 5:**
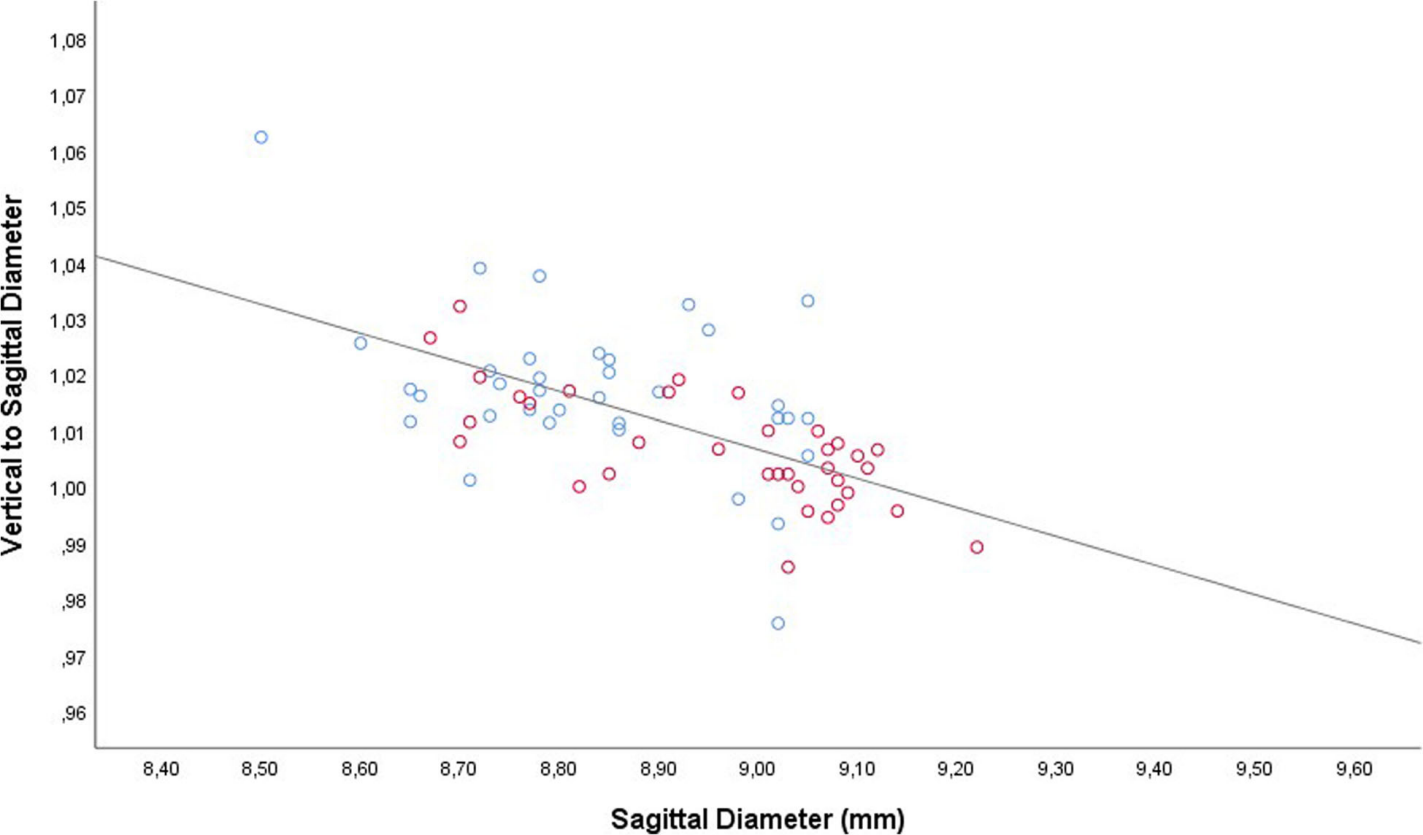
Scattergram showing the correlation between the sagittal globe diameter and the ratio of the vertical to sagittal globe diameter in young guinea pigs undergoing unilateral lens-induced myopization in the right eyes (study group), with the left eyes remaining untouched (control group); equation of the regression line: Ratio of Vertical to Sagittal Globe Diameter = − 0.05 x Sagittal Diameter (mm) + 1.47; regression coefficient r^2^ = 0.37; *P* < 0.001. Blue circles: Control group; Red circles: Myopia group

Correspondingly, the ratio of the horizontal to sagittal globe diameter decreased significantly with longer sagittal globe diameter, from a value of 1.06 for eyes with an axial length of 8.40 mm to a value of 1.00 for eyes with an axial length of 9.55 mm (equation of the regression line: Ratio of Horizontal to Sagittal Globe Diameter = − 0.05 x Sagittal Diameter (mm) + 1.43; regression coefficient r^2^ = 0.18; *P* < 0.001) (Fig. 4). In a similar manner, the ratio of the vertical to sagittal globe diameter decreased from a value of 1.04 for eyes with an axial length of 8.40 mm to a value of 1.00 for eyes with an axial length of 9.15 mm (equation of the regression line: Ratio of Vertical to Sagittal Globe Diameter = − 0.05 x Sagittal Diameter (mm) + 1.47; regression coefficient *r*^2^ = 0.37; *P* < 0.001) (Fig. 5).

## Discussion

While the non-standardized regression coefficient for the association between the vertical globe diameter and the horizontal globe diameter was close to 1.0, indicating that both diameters increased by a ratio of approximately 1 to 1, the non-standardized regression coefficients for the relationships of both diameters with the sagittal diameter were 0.64 (relationship horizontal to sagittal diameter) and 0.55 (relationship vertical to sagittal diameter). It indicated that for each millimeter axial elongation the horizontal globe diameter increased by 0.64 mm (95% CI: 0.44, 0.83) and the vertical diameter by 0.55 mm (95% CI: 0.41, 0.69). Correspondingly, the ratio of the horizontal or vertical globe diameter to the sagittal globe diameter decreased with longer axial length: While in shorter eyes, the axial length was shorter than the coronal diameters, all three diameters were almost equal in length in longer globes (Figs. 4, 5). It suggested that the enlargement of the globes had taken place mostly in sagittal direction. The axial elongation in guinea pigs was thus similar to the axial elongation of myopic eyes in humans.

In humans, axial myopia is associated with an elongation of the eye in the sagittal direction. As early as 1899, Heine described that myopic eyes showed an enlargement of the globe mostly posterior to the globe [1]. It resulted in a change of the ocular shape from a sphere-like form in emmetropic eyes to an axially elongated shape in the myopic eyes. Using high-resolution magnetic resonance imaging, Moriyama and colleagues reported that that the most enlarged part of highly myopic human globes with myopic retinopathy was orientated along the central sagittal axis in about 80% of the eyes and that it was located slightly inferior to the central axis in the remaining eyes [16]. Histomorphometric investigations of enucleated human globes and clinical studies of myopic patients have shown that this axial myopic elongation led to changes located mostly at the posterior pole and to a lower degree in the region of the ocular equator, while the region anterior to the ora serrata demonstrated only minor changes [14, 17–20]. Correspondingly, the axial myopia-associated thinning of the sclera and choroid was most profound at the posterior pole and least marked at, or anterior to, the equator [17–20]. The thickness of the sclera in the pars plana region in human globes did not differ markedly between eyes with primary high axial myopia and emmetropic eyes. In human eyes with secondary high axial myopia due to congenital glaucoma however, the sclera was markedly thinned in all regions, parallel to an enlargement and thinning of the cornea [21]. Correspondingly, a recent study on the ocular dimensions of enucleated eyes of adult patients showed that in eyes with an axial length of equal to or less than 24 mm, the horizontal diameter and the vertical diameter increased by 0.44 mm and 0.51 mm, respectively, for each millimeter increase in axial diameter, while in eyes with an axial length of more than 24 mm, the horizontal diameter and the vertical diameter increased by 0.19 mm and 0.21 mm for each mm enlargement in axial diameter [3]. These measures agreed with the concept of a mostly axial elongation of myopic eyes, and that a higher degree of myopic elongation of the eyes was associated with a decreasing enlargement of the eyes in the coronal plane.

The results of our study indicated that also in guinea pigs increasing axial elongation was associated with a reduction in the increase in the coronal globe diameters (Figs. 4, 5). One may assume that the model of lens-induced axial elongation in guinea pigs led to a similar change in the globe shape as in humans. The lower ratio of horizontal or vertical globe enlargement in relationship to the sagittal enlargement in the human eyes as compared to the guinea pig eyes of the present study might have been due to differences between both studies in the amount of axial elongation. While in the present study, the guinea pig eyes were only slightly elongated, the histological study on human eyes included extremely elongated globes with an axial length of more than 35 mm. In addition, both studies differed in the age of the study populations.

As in human eyes, the eyes of the guinea pigs showed an elongation mostly in the sagittal direction. It suggested that the globe wall had enlarged predominantly in the equatorial region. Interestingly, the sensory region for detecting a defocus of the image on the retina has been discussed to be located in the midperiphery of the fundus in the equatorial to retro-equatorial region [22–25]. It could indicate that the afferent arm and the efferent arm of the feed-back mechanism of the process of emmetropization and myopization are located in the same region.

The animal model of lens-induced myopization has been well established for many years. In the current study, the difference in axial length between the experimental group and the control group was statistically significant (8.96 ± 0.15 mm versus 8.84 ± 0.14 mm; *P* = 0.001), however relatively small. The difference of 0.12 mm in axial length between both groups compared well with the observations made in other investigations with a similar study design. In one of these studies, the difference between eyes of young guinea pigs with goggles and those without goggles was 8.88 ± 0.08 mm (study group) and 8.67 ± 0.09 mm (control group) (*P* = 0.002) [13]. In another investigation, the study and control group differed in axial length from 8.93 ± 0.06 mm to 8.76 ± 0.03 mm [26]. In guinea pigs with unilateral lens-induced axial elongation, axial length differed between both eyes from 8.93 ± 0.06 mm to 8.73 ± 0.03 mm [26]. A difference similar to the one found in our study was reported in a study by Xiao and colleagues in which guinea pigs with lens-induced myopization (7.89 ± 0.06 mm) and guinea pigs of a control group (7.75 ± 0.04 mm) differed in axial length by 0.14 mm [27].

Potential limitations of our study should be considered. First, the globe diameters were measured with the globes intact. The measurements of these external globe diameters thus included the thickness of the retina, choroid and sclera. The difference between the external diameter and the internal diameter may be of importance in particular for the sagittal diameter since the length of the optical axis is measured as distance from the corneal apex to the level of the photoreceptor outer segments/retinal pigment epithelium only. The length of the optical axis as compared to the external globe diameter is the main parameter for the process of emmetropization/myopization. Second, the control group included eyes wearing goggles with no refractive power, so that these eyes were not completely “naïve” in the sense of no intervention at all. Third, refractometric data were not provided so that the study basically assessed the influence of wearing goggles (with a refractive power of − 10 diopters) on the ocular globe diameters. Fourth, the globe shape and the diameters depend on the actual intraocular pressure which was not measured just prior to the measurements. Since however the globe diameters were assessed shortly after enucleation, all globes were measured at roughly similar conditions.

## Conclusions

In conclusion, for each millimeter axial elongation in young guinea pigs the horizontal globe diameter increased by 0.64 mm (95% CI: 0.44, 0.83) and the vertical diameter by 0.55 mm (95% CI: 0.41, 0.69), indicating that the globe enlargement occurred predominantly in the sagittal direction. The axial elongation in young guinea pigs led to a similar relative change in ocular shape as in humans.

## Abbreviations

CI: confidence interval
Fig: Figure
